# Relationship between bisphenol A and autoimmune thyroid disease in women of childbearing age

**DOI:** 10.3389/fendo.2024.1333915

**Published:** 2024-01-29

**Authors:** Ning Yuan, Jianbin Sun, Xin Zhao, Wei Li

**Affiliations:** ^1^ Department of Endocrinology, Peking University International Hospital, Beijing, China; ^2^ Department of General Surgery, Peking University International Hospital, Beijing, China

**Keywords:** autoimmune thyroid disease, thyroglobulin antibody, bisphenol A, anti-thyroid peroxidase antibody, euthyroidism

## Abstract

**Background:**

Autoimmune thyroid disease (AITD) is the main cause of hypothyroidism in women of childbearing age. Bisphenol A (BPA) is an environmental factor affecting AITD. This study aims to investigate relationship between BPA and AITD in women of childbearing age, thereby contributing novel evidence for the prevention of hypothyroidism in this specific demographic.

**Methods:**

A total of 155 women of childbearing age were enrolled in this study, including the euthyroid group comprised 60 women with euthyroidism and thyroid autoantibodies negativity and the AITD group consisted of 95 women with euthyroidism and at least one thyroid autoantibody positivity. The general information, thyroid function, thyroid autoantibodies, and thyroid ultrasound results of the two groups of women of childbearing age were recorded. Urinary BPA and urinary BPA/creatinine were detected. The difference of BPA levels between the two groups was compared. logistic regression was used to analyze the correlation between BPA and AITD.

**Results:**

The proportion of multiparous and serum thyroid stimulating hormone levels were significantly higher in the AITD group compared to the euthyroid group. Logistic regression analysis revealed that BPA levels did not exhibit a statistically significant association with AITD. Spearman correlation analysis revealed a statistically significant correlation between BPA and urinary iodine levels (r=0.30, P < 0.05), as well as a correlation between urinary BPA and free tetraiodothyronine (FT4) levels (r=0.29, P < 0.05).

**Conclusion:**

This study revealed a correlation between urinary BPA levels and FT4 levels. However, it did not establish a relationship between BPA and AITD in women of childbearing age.

## Background

Bisphenol A (BPA, 4,4´-isopropylidenediphenol) is a key component in the production of polycarbonate, epoxy resins, and various polymer materials. It is commonly utilized as an additive in food containers, medical devices, plastics, and various other products (1). BPA can influence autoimmunity directly and indirectly, including exogenous estrogen effects, interference with T cell aggregation, elevated prolactin levels, disruption of cytochrome P450, and the generation of reactive oxygen species (2–4). Autoimmune thyroid disease (AITD) stands as a prominent cause of hypothyroidism among women of childbearing age (5). The impact of hypothyroidism on women of childbearing age can have far-reaching consequences. Hypothyroidism may disrupt the menstrual cycle and ovulation, potentially leading to infertility, while during pregnancy, it heightens the risk of adverse outcomes like miscarriage, preterm birth, premature rupture of membranes, and eclampsia (6). AITD is primarily characterized by the presence of autoantibodies against thyroid antigens in the bloodstream, including thyroglobulin antibody (TGAb), anti-thyroid peroxidase antibody (TPOAb), thyrotropin receptor (TRAb), and more (7). The precise pathogenesis of AITD remains elusive, but both genetic and environmental factors are recognized as pivotal contributors to AITD (8). AITD is more prevalent among women than men (9). Therefore, it is extremely imperative to explore the pathogenesis of AITD and prevent hypothyroidism caused by AITD in women of childbearing age. However, the available literature on BPA’s association with AITD is scarce and exhibits divergent views. One study examining the correlation between serum BPA and thyroid autoantibodies (10) showed BPA was independently associated with TPOAb positivity. In Chinese women, higher urinary BPA concentration was associated with increased risk of thyroid nodules, specifically in individuals with positive thyroid autoantibodies (11). Contrarily, another study reported no association between serum TPOAb and TGAb and BPA (12). Consequently, while BPA is acknowledged as an environmental factor impacting AITD, its effect on AITD in women of childbearing age remains a subject of controversy. Therefore, the primary objective of this study is to explore the relationship between BPA and AITD in women of childbearing age, thereby contributing novel evidence for the prevention of hypothyroidism in this specific demographic.

## Methods

### Study population

The cross-sectional clinical study was conducted from January 2019 to January 2020 at the Department of Endocrinology, Peking University International Hospital. The study received approval from the Committee on Bioethics at Peking University International Hospital (Ethics Batch Number: 2018-061(BMR)), and written informed consent was obtained from all participants. Inclusion criteria were defined as follows: (a) Female participants aged 20-45 years; (b) AITD group: thyroid autoantibody positive (≥1 thyroid autoantibody) with euthyroidism. Euthyroid group: thyroid autoantibody negative with euthyroidism; (c) No history of hyperthyroidism, hypothyroidism, thyroid surgery, I131 radiotherapy, or the use of thyroid hormones or antithyroid drugs; (d) Willingness to participate and provision of informed consent. Exclusion criteria encompassed: (a) Individuals with severe liver, kidney, heart, and other vital organ failure; (b) Those with concomitant autoimmune diseases except AITD; (c) Individuals taking medications such as amiodarone or iodine contrast agents that could influence thyroid function. A total of 155 women attending the Endocrinology Department of Peking University International Hospital voluntarily enrolled in this study. The euthyroid group comprised 60 women with euthyroidism and thyroid autoantibodies negativity. The AITD group consisted of 95 women with euthyroidism and at least one thyroid autoantibody positivity. This study was registered on ClinicalTrials.gov (Identifier: NCT03932487). All women completed a questionnaire covering their medical history related to thyroid and autoimmune diseases, family history of thyroid disorders, age of menarche, reproductive history, usage of iodized salt, and education level. Additional data recorded included age, height, weight, and blood pressure. The body-mass index (BMI) was calculated as the ratio of weight in kilograms to height in meters squared.

### Thyroid function tests

Serum samples were collected, and the following parameters were measured using a COBAS Elesys 601 immunoassay analyzer (Roche Diagnostics, Switzerland) in the clinical laboratory of Peking University International Hospital: TSH (Thyroid-Stimulating Hormone), FT4 (Free Thyroxine), FT3 (Free Triiodothyronine), TT4 (Total Thyroxine), TT3 (Total Triiodothyronine), TGAb, TPOAb, and TRAb. Positivity thresholds were defined as a TPOAb titer of 34 IU/mL or more, a TGAb titer of 115 IU/mL or more, and a TRAb titer of 115 IU/mL.

### BPA

Serum BPA levels were determined through competitive enzyme-linked immunosorbent assay (ELISA) (ABCAM, Cambridge, UK) with a detection limit of 0.3 pg/ml. Intra- and inter-assay precision were 7.0% and 13.6%, respectively. Based on the quartile distribution of BPA levels, the patients were categorized into four groups: Q1 group (< Q25), Q2 group (Q25-Q50), Q3 group (Q50-Q75), and Q4 group (> Q75).

### Ultrasonography

Thyroid gland ultrasonography was conducted by a skilled sonographer. Patients were positioned in the supine posture, ensuring full exposure of the neck for comprehensive examination of the thyroid gland and associated lymph nodes in the drainage region. Gray scale ultrasound was employed to assess various aspects, including the number, location, shape, boundaries, internal echoes, presence of silent halos, calcification, and lesion size. Abnormal lymph nodes in the drainage area were scrutinized for their shape, number, internal echoes, and measured for size. To examine the blood flow characteristics of lesions and lymph nodes in the drainage area, color Doppler ultrasound was utilized. Scanning of the neck involved sagittal, transverse, and oblique sections to optimize the visualization of both thyroid gland lobes and the isthmus.

### Sample size calculation

Drawing from previous literature, urinary BPA levels in the AITD group were estimated to be 7.26 μg/L, with a standard deviation(SD) of 5.78 μg/L. In contrast, the euthyroid group exhibited estimated levels of 4.58 μg/L, with a SD of 3.15 μg/L. To perform the statistical analysis, we set the significance level (α) at 0.05 (bilateral), while the desired statistical power (β) was set at 0.20. Utilizing PASS software, a sample size of 96 cases was calculated as the minimum requirement for the study. Accounting for an anticipated 10% attrition rate among subjects, the total sample size was determined to be 107 cases.

### Statistical analysis

Statistical analyses were conducted using SPSS version 17.0 (IBM, USA). Continuous data following a normal distribution were presented as mean ± SD, while non-normally distributed data were expressed as median (interquartile range [IQR]). Categorical data were represented as counts and percentages. Differences between the euthyroid group and the AITD group were assessed using the t-test or Mann-Whitney U test for continuous data and the Fisher’s exact test for categorical data. A multivariate logistic regression model was applied to investigate the association between BPA levels and AITD, TPOAb positivity and TGAb positivity. Spearman correlation was used to determine the correlation between urinary BPA and thyroid function, thyroid autoantibodies, and urinary iodine. A significance level of P < 0.05 was deemed statistically significant.

## Results

### Characteristics of participants

**Table 1** presents the characteristics of the women participating in this study. The study included 60 women in the euthyroid group and 95 women in the AITD group. Notably, the AITD group exhibited a higher proportion of multiparous women compared to the euthyroid group (68.4% vs. 48.3%, P < 0.05). However, there were no significant differences observed in terms of age, BMI, education level, usage of iodized salt, and age of menarche between the two groups.

**Table 1:** Clinical characteristics in euthyroid group and AITD group.

Characteristic	Euthyroid group (n = 60)	AITD group (n = 95)	*P* Value
Age (years), median (Q25, Q75)	31 (28.5,36)	31 (28,34)	0.27
BMI (kg/m^2^), median (Q25, Q75)	21.50 (19.76, 24.47)	21.35 (19.86,23.71)	0.623
Education level (%)			
None or primary only	2 (3.3%)	12 (12.6%)	0.141
Secondary education	36 (60%)	53 (55.8%)	
Higher education	22 (36.7%)	30 (31.6%)	
Consumption of salt, n(%)			
Iodized salt	51 (85%)	81 (85.3%)	0.643
Non-iodized salt	4 (6.7%)	9 (9.5%)	
Mixed	5 (8.3%)	5 (5.3%)	
Parity (%)			
primipara	31 (51.7%)	30 (31.6%)	0.013
multipara	29 (48.3%)	65 (68.4%)	
Age of menarche, (years),median (Q25, Q75)	13 (12,13.7)	13 (12,14)	0.24

*P < 0.05.

BMI, body mass index; AITD, autoimmune thyroid disease.

**Table 2** illustrates the comparison of thyroid function, urinary iodine, urinary BPA levels, and the prevalence of thyroid nodules between the two groups. Serum TSH levels were significantly higher in the AITD group compared to the euthyroid group (median TSH 2.18 uIU/ml, IQR [1.55-3.28] uIU/ml vs. 1.83 uIU/ml, IQR [1.48-2.34] uIU/ml, P < 0.05). However, there were no significant differences observed between the two groups in terms of urinary iodine levels, urinary BPA levels, and the prevalence of thyroid nodules.

**Table 2:** The comparison of thyroid function, urinary iodine, urinary BPA levels, and the prevalence of thyroid nodules between euthyroid group and AITD group.

Compound	Euthyroid group (n = 60)	AITD group (n = 95)	*P* Value
FT4 (pmol/L),median (Q25, Q75)	16.30 (15.00, 18.00)	15.80(14.6,17.30)	0.272
FT3 (pmol/L), median (Q25, Q75)	4.50 (4.17, 5.00)	4.60 (4.20,5.10)	0.608
TT3(nmol/L), median (Q25, Q75)	1.60 (1.50, 1.90)	1.60 (1.40,1.90)	0.802
TT4(nmol/L), median (Q25, Q75)	100.30(86.80, 111.80)	99.40(87.20,112.00)	0.998
TSH (uIU/ml),median (Q25, Q75)	1.83 (1.48, 2.34)	2.18 (1.55, 3.28)	0.016
Urine iodine (μg/L),median (Q25, Q75)	134.50(72.00, 204.00)	136.00(74.25,209.25)	0.688
Urine iodine/urine creatinine(μg/L), median (Q25, Q75)	20.89 (12.88, 31.24)	19.94 (11.26,29.60)	0.464
Urine BPA(μg/L), median (Q25, Q75)	1.13 (0.20, 3.47)	1.73 (0.30,8.16)	0.454
Urine BPA/urine creatinine(μg/L), median (Q25, Q75)	0.32 (0.08, 0.88)	0.29 (0.10,1.18)	0.849
Thyroid nodule, n (%)	18 (30%)	20 (21.1%)	0.207

*P < 0.05.

AITD, autoimmune thyroid disease; FT4, free thyroxine; FT3, free triiodothyronine; TT4, total thyroxine; TT3, total triiodothyronine; TSH, thyroid-stimulating hormone; BPA, bisphenol A.

### Incidence of AITD in groups with different levels of BPA

**Figure 1:**
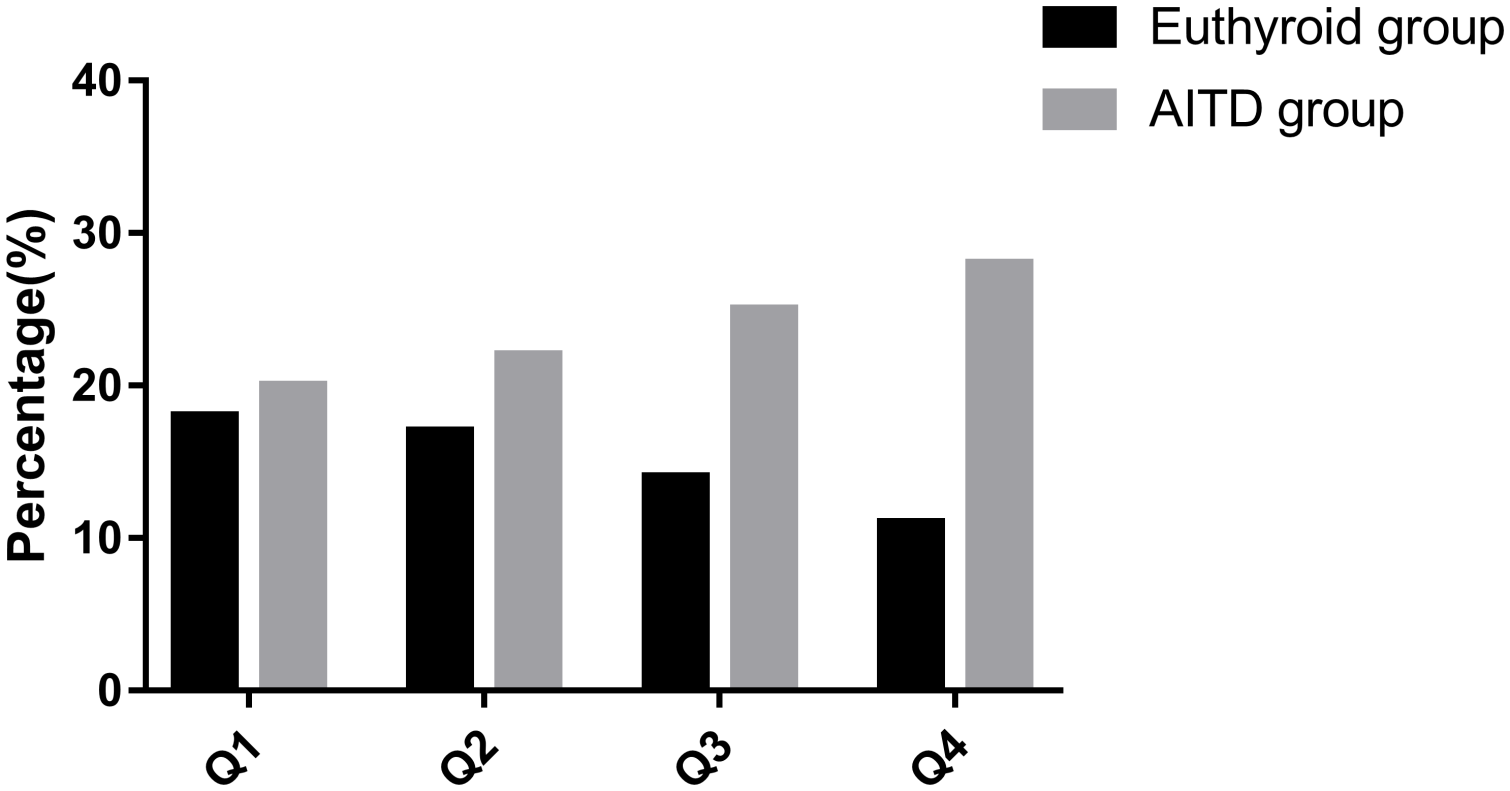
Incidence of AITD in groups with different levels of BPA. AITD, autoimmune thyroid disease.

Patients were categorized into four groups, Q1 to Q4, based on quartiles of BPA levels. The incidence of AITD in these groups was as follows: 52.6%(n=20) in Q1, 56.4%(n=22) in Q2, 64.1%(n=25) in Q3, and 71.8%(n=20) in Q4 (**Figure 1**). It’s worth noting that the differences among these groups were not statistically significant (P > 0.05).

### Multivariate logistic regression analysis

The logistic regression analysis, considering AITD, TPOAb positivity, and TGAb positivity as categorical dependent variables, demonstrated that BPA levels did not show a statistically significant association with any of these variables. This observation persisted even after adjusting for maternal age, BMI, parity, usage of iodized salt, and age of menarche, as detailed in **Table 3**.

**Table 3:** Multivariate logistic regression analysis.

BPA	AITD	TGAb positivity	TPOAb positivity
	OR(95%CI)	OR(95%CI)	OR(95%CI)
Q1	Ref	Ref	Ref
Q2	1.167 (0.257,5.293)	1.556 (0.340,7.11)	1.111 (0.213,5.802)
Q3	1.5 00(0.344,6.532)	1.524 (0.352,6.601)	2.857 (0.612,13.336)
Q4	3 .000(0.562,16.013)	1.867 (0.392,8.894)	2.500(0.495,12.635)

BPA, bisphenol A; AITD, autoimmune thyroid disease; TG, thyroglobulin antibody; TPO, anti-thyroid peroxidase antibody.

### The correlation between urinary BPA and thyroid function, thyroid autoantibodies, and urinary iodine

Spearman correlation analysis was employed to investigate the correlations between urinary BPA levels and thyroid function, thyroid autoantibodies, and urinary iodine. The analysis revealed a statistically significant correlation between BPA and urinary iodine levels (r=0.30, P < 0.05), as well as a correlation between urinary BPA and FT4 levels (r=0.29, P < 0.05).

## Discussion

This study investigated the association between BPA levels and AITD and compared its findings to previous research (**Table 4**). However, the association between elevated BPA level and AITD was not found in this study, and some previous studies have shown similar results. For example, one study found no significant association between serum TPOAb and TGAb with urinary phthalate metabolites and BPA. However, it observed that thyroid autoantibody status influenced the relationship between certain phthalates and thyroid hormones (12). Another study in lab animals found that BPA could affect the expression of thyroid hormone synthesis related genes in FRTL-5 cells of thyroid follicles of rats without affecting TPO activity (13).

**Table 4:** Summary of research on the correlation between BPA, thyroid autoantibodies, and thyroid function.

Author	Time	Country	Study population	Outcomes	Correlation	OR/RR, 95%CI
Choi S (12)	2015-2017	Korea	Adult	TPOAb,TGAb	No	
Wu Y (13)	2016	USA	Rat	TPOAb	No	
Chailurkit L-o (10)	2009	Thailand	≥15 years old	TPOAb, TGAb	Yes	TGAb 1.13 (1.00-1.27) TPOAb1.34 (1.21-1.50)
Li L (11)	2017-2018	China	Adult	TPOAb, TGAb	Yes	In AITD group, BPA and thyroid nodule risk was near linear
Yuan S (14)	before 23rd May 2022	–	Adult	FT4, TSH	Yes	Male FT4−0.027 (−0.030∼−0.024), TSH −0.058 (−0.111∼−0.004) Female FT4 0.006 (0.003–0.008).
Jang Y (15)	2015-2017	Korea	6-year-old children	FT4, TT3, TSH	No	
Yue B (16)	2017	China	Adult	FT4, FT3, TT4, TT3, TSH	No	
Milczarek-Banach J (17)	2017-2019	Poland	Female	TSH FT4	No	

FT4, free thyroxine; FT3, free triiodothyronine; TT4, total thyroxine; TT3, total triiodothyronine; TSH, thyroid-stimulating hormone; TG, thyroglobulin antibody; TPO, anti-thyroid peroxidase antibody.

However, there were some studies that were inconsistent with the results of this study. A research (10) on the association between serum BPA and thyroid autoantibodies showed that BPA was independently associated with TPOAb positivity. There was a trend toward a significant increase in TgAb and TPOAb positive subjects as the BPA quartile increased, especially in the highest quartile. In Chinese women with thyroid autoantibodies positivity, higher urinary BPA concentrations were linearly associated with increased risk of thyroid nodules (11).

Furthermore, this study observed a correlation between urinary BPA and FT4 levels (r=0.29, P < 0.05), which aligns with findings in previous research. A systematic review and meta-analysis (14) on BPA exposure and thyroid dysfunction in adults revealed a positive correlation between BPA concentration and FT4 in females, and the pooled correlation coefficient was 0.006 (95%CI = 0.003-0.008). Nonetheless, there are studies that deviate from the results of this study. A study in 6-year-old children in Korea did not find a statistically significant relationship between urinary BPA and thyroid hormone concentrations (15). A cross-sectional study examined the associations of multiple chemicals with thyroid hormones among adults in China found that BPA was not associated with thyroid function (16). Similarly, a study on BPA analogues and thyroid function and thyroid volume in women of childbearing age showed no correlation between BPA and thyroid function (17).

Differences in BPA levels may stem from the environmental conditions of the study participants, the methods used for BPA detection, and the characteristics of the study populations. BPA levels are influenced by environmental factors, and various detection methods, such as spectroscopy, chromatography, ELISA, and mass spectrometry, may yield different results. Moreover, the stability of phenolic compounds can vary depending on sample types and storage methods. Additionally, this study predominantly included women of childbearing age, which differs from the previous study populations.

It’s important to note that BPA can affect autoimmune and thyroid hormones through various mechanisms. BPA binds to estrogen receptors to enhance the immune response by affecting the differentiation and function of regulatory T cells and Th1/Th2 polarization (18, 19). In naive condition, administered BPA disturbed antigen specific immune responses by reducing interleukin (IL)-2, 4, and interferon (IFN)-gamma secretion, and increasing the IgA and IgG2a production (20). BPA can affect thyroid hormone gene regulation. In vertebrate experiments, microarray analysis showed that BPA antagonized the regulation of most T(3) response genes, suggesting that BPA mainly affects the thyroid hormone T(3) signaling pathway and thus affects the development of vertebrates *in vivo (* 21). BPA was found to antagonize the action of T (3) at the transcriptional level: BPA could replace T (3) in TRs and recruit a transcriptional suppressor, thereby inhibiting gene expression, and BPA inhibited T (3) stimulated transcriptional activity in a dose-dependent manner. BPA can antagonize thyroid hormone effect through β-TRs and affect thyroxine level (22). Protein disulfide isomerase (PDI) regulates gene expression induced by T3, while BPA disrupts thyroid hormone function in somsterin-3 cells by inhibiting PDI action (23).

This study’s strengths included the inclusion of women of childbearing age from the same area, eliminating gender and environmental bias. Moreover, it focused on the contentious topic of BPA and autoimmune thyroid diseases. This study, however, has several limitations that should be acknowledged. Firstly, the sample size is relatively small, and the study design is cross-sectional. Nevertheless, we enhanced the study’s reliability by implementing rigorous data collection standardization, improving data analysis accuracy, and employing scientifically robust research methods. Additionally, BPA detection was performed using ELISA rather than the more advanced liquid chromatography-tandem mass spectrometry (LC-MS/MS). It’s noteworthy that in this study, participants were categorized into four groups based on BPA quartiles, serving as the factor variable for ordered classification.

## Conclusions

This study revealed a correlation between urinary BPA levels and FT4 levels. However, it did not establish a relationship between BPA and AITD in women of childbearing age.

## Data availability statement

The raw data supporting the conclusions of this article will be made available from the corresponding author on reasonable request.

## Ethics statement

The studies involving humans were approved by Ethics Committee of Peking University International Hospital. Written informed consent was obtained from each participant before enrolment in this study before samples collection. The studies were conducted in accordance with the local legislation and institutional requirements. The participants provided their written informed consent to participate in this study.

## Author contributions

NY: Data curation, Formal analysis, Funding acquisition, Investigation, Project administration, Writing – original draft. WL: Supervision, Writing – review & editing. JS: Data curation, Resources, Writing – review & editing. XZ: Data curation, Resources, Writing – review & editing.
